# Draft Genome Sequence of *Lactobacillus johnsonii* Strain 16, Isolated from Mice

**DOI:** 10.1128/genomeA.01141-15

**Published:** 2015-10-08

**Authors:** Keren Buhnik-Rosenblau, Yael Danin-Poleg, Sharona Elgavish, Yechezkel Kashi

**Affiliations:** a Faculty of Biotechnology and Food Engineering, Technion-Israel Institute of Technology, Haifa, Israel; b Info-CORE, Bioinformatics Unit of the I-CORE Computation Center at the Hebrew University and Hadassah, The Institute for Medical Research Israel-Canada, The Hebrew University-Hadassah Medical School, Jerusalem, Israel

## Abstract

Here, we report the genome sequence of *Lactobacillus johnsonii*, a member of the gut lactobacilli. This draft genome of *L. johnsonii* strain 16 isolated from C57BL/6J mice enables the identification of bacterial genes responsible for host-specific gut persistence.

## GENOME ANNOUNCEMENT

*Lactobacillus johnsonii* belongs to the lactic acid bacteria. It inhabits the gut of several hosts (1–6) and includes strains with probiotic activity (7–11). *L. johnsonii* levels were shown to be significantly higher in fecal samples of C57BL/6J mice than those of BALB/c. It was further suggested that the host genetics has a major effect on the persistence of *L. johnsonii* strain 16 in the gut of C57BL/6J mice (12). Here, we describe the draft genome sequence of *L. johnsonii* strain 16 isolated from a fecal sample of C57BL/6J mice in 2007, grown at Technion, Israel, and subjected to whole-genome shotgun sequencing.

Two different libraries, with average insert sizes of 330 bp and 3,700 bp, were prepared and sequenced using Illumina Genome Analyzer IIx, generating 5,908,676 31-bp paired-end reads and 4,615,910 31-bp mate-pair past-filtered reads, with a coverage of 183×. The reads of the two libraries were *de novo* assembled simultaneously with different insert lengths with Velvet 0.7.54 (13), generating 210 contigs of >100 bp using reads with a minimum quality of 30 for each base. The assembly covers 1,794,984 bp, with an *N*_50_ of 34,798 bp and a longest segment of 118,507 bp. Mapping was done to validate the *de novo* assembly using MAQ 0.7.1 and BWA 0.5.8c (14), with a maximum of two differences from the reference sequence per read. Of the single reads, 95.6% were mapped to the assembly, and 80% of the single reads were mapped to the *L. johnsonii* NCC533 genome (15), covering 84.8% of the genome. In parallel, the 210 contigs were contiguated (aligned, ordered, and oriented), using ABACAS (16), to the *L. johnsonii* NCC 533 chromosome (15), which served as a reference genome. One scaffold that splits into 133 contigs was produced, with a total length of 1,491,125 bp, compared to 1,992,676 bp of the *L. johnsonii* NCC 533 genome. Seventy-seven contigs of the original 210 contigs could not be aligned to that reference genome. This implies that the human isolate *L. johnsonii* NCC 533 may not be a proper reference genome for the assembly of the mouse isolate *L. johnsonii* strain 16. Therefore, we relied on the *de novo* assembly, in which contigs <200 bp were removed.

The draft genome of *L. johnsonii* strain 16 consists of 156 segments covering 1.78 Mbp (34.6% G+C content). A total of 1,703 coding sequences (CDSs), 49 pseudogenes, 4 rRNAs, 42 tRNAs, and 1 noncoding RNA (ncRNA) were predicted and annotated by the NCBI Prokaryotic Genome Annotation Pipeline (17).

The whole-genome sequence of *L. johnsonii* strain 16 might lead to the discovery of bacterial genes that may be involved in bacterial persistence in the gut. In a previous study, we showed a phylogenetic separation among *L. johnsonii* isolates associated with the taxonomic classification of their hosts, indicating coevolution of the host and its gut bacteria (18). Thus, the gathered information on both host and bacterial genes involved in bacterial gut persistence call for the development of probiotic products specifically oriented to the consumer’s genetics as part of a personalized medicine approach.

### Nucleotide sequence accession numbers.

This whole-genome shotgun project has been deposited at DDBJ/EMBL/GenBank under the accession no. LIGY00000000. The version described in this paper is version LIGY00000000.1.

## References

[1] Kim SY, Adachi Y 2007 Biological and genetic classification of canine intestinal lactic acid bacteria and bifidobacteria. Microbiol Immunol 51:919–928. doi:10.1111/j.1348-0421.2007.tb03983.x. 17951981

[2] Peña JA, Li SY, Wilson PH, Thibodeau SA, Szary AJ, Versalovic J 2004 Genotypic and phenotypic studies of murine intestinal lactobacilli: species differences in mice with and without colitis. Appl Environ Microbiol 70:558–568. doi:10.1128/AEM.70.1.558-568.2004. 14711688 PMC321283

[3] Stephenson DP, Moore RJ, Allison GE 2009 Comparison and utilization of repetitive-element PCR techniques for typing *Lactobacillus* isolates from the chicken gastrointestinal tract. Appl Environ Microbiol 75:6764–6776. doi:10.1128/AEM.01150-09. 19749057 PMC2772419

[4] Carina Audisio M, Torres MJ, Sabaté DC, Ibarguren C, Apella MC 2011 Properties of different lactic acid bacteria isolated from *Apis mellifera* L. bee-gut. Microbiol Res 166:1–13. doi:10.1016/j.micres.2010.01.003. 20116222

[5] Korhonen JM, Sclivagnotis Y, von Wright A 2007 Characterization of dominant cultivable lactobacilli and their antibiotic resistance profiles from faecal samples of weaning piglets. J Appl Microbiol 103:2496–2503. doi:10.1111/j.1365-2672.2007.03483.x. 18045434

[6] Dec M, Puchalski A, Urban-Chmiel R, Wernicki A 2014 Screening of *Lactobacillus* strains of domestic goose origin against bacterial poultry pathogens for use as probiotics. Poult Sci 93:2464–2472. doi:10.3382/ps.2014-04025. 25104766

[7] du Toit M, Franz CM, Schillinger U, Haberer P, Warlies B, Ahrens F, Holzapfel WH 1998 Characterisation and selection of probiotic lactobacilli for a preliminary minipig feeding trial and their effect on serum cholesterol levels, faeces pH and faeces moisture content. Int J Food Microbiol 40:93–104. doi:10.1016/S0168-1605(98)00024-5. 9600615

[8] La Ragione RM, Narbad A, Gasson MJ, Woodward MJ 2004 *In vivo* characterization of *Lactobacillus johnsonii* FI9785 for use as a defined competitive exclusion agent against bacterial pathogens in poultry. Lett Appl Microbiol 38:197–205. doi:10.1111/j.1472-765X.2004.01474.x. 14962040

[9] Lai KK, Lorca GL, Gonzalez CF 2009 Biochemical properties of two cinnamoyl esterases purified from a *Lactobacillus johnsonii* strain isolated from stool samples of diabetes-resistant rats. Appl Environ Microbiol 75:5018–5024. doi:10.1128/AEM.02837-08. 19502437 PMC2725488

[10] Vizoso Pinto MG, Schuster T, Briviba K, Watzl B, Holzapfel WH, Franz CM 2007 Adhesive and chemokine stimulatory properties of potentially probiotic *Lactobacillus* strains. J Food Prot 70:125–134. 17265871 10.4315/0362-028x-70.1.125

[11] Van Coillie E, Goris J, Cleenwerck I, Grijspeerdt K, Botteldoorn N, Van Immerseel F, De Buck J, Vancanneyt M, Swings J, Herman L, Heyndrickx M 2007 Identification of lactobacilli isolated from the cloaca and vagina of laying hens and characterization for potential use as probiotics to control *Salmonella* Enteritidis. J Appl Microbiol 102:1095–1106. doi:10.1111/j.1365-2672.2006.03164.x. 17381753

[12] Buhnik-Rosenblau K, Danin-Poleg Y, Kashi Y 2011 Predominant effect of host genetics on levels of *Lactobacillus johnsonii* bacteria in the mouse gut. Appl Environ Microbiol 77:6531–6538. doi:10.1128/AEM.00324-11. 21803912 PMC3187140

[13] Zerbino DR, Birney E 2008 Velvet: algorithms for *de novo* short read assembly using de Bruijn graphs. Genome Res 18:821–829. doi:10.1101/gr.074492.107. 18349386 PMC2336801

[14] Li H, Durbin R 2010 Fast and accurate long-read alignment with Burrows-Wheeler transform. Bioinformatics 26:589–595. doi:10.1093/bioinformatics/btp698. 20080505 PMC2828108

[15] Pridmore RD, Berger B, Desiere F, Vilanova D, Barretto C, Pittet AC, Zwahlen MC, Rouvet M, Altermann E, Barrangou R, Mollet B, Mercenier A, Klaenhammer T, Arigoni F, Schell MA 2004 The genome sequence of the probiotic intestinal bacterium Lactobacillus johnsonii NCC 533. Proc Natl Acad Sci U S A 101:2512–2517. doi:10.1073/pnas.0307327101. 14983040 PMC356981

[16] Assefa S, Keane TM, Otto TD, Newbold C, Berriman M 2009 ABACAS: algorithm-based automatic contiguation of assembled sequences. Bioinformatics 25:1968–1969. doi:10.1093/bioinformatics/btp347. 19497936 PMC2712343

[17] Tatusova TD, DiCuccio M, Badretdin A, Chetvernin V, Ciufo S, Li W 2013 Prokaryotic Genome Annotation Pipeline. The NCBI handbook, 2nd ed. National Center for Biotechnology Information, Bethesda, MD. http://www.ncbi.nlm.nih.gov/books/NBK174280/.

[18] Buhnik-Rosenblau K, Matsko-Efimov V, Jung M, Shin H, Danin-Poleg Y, Kashi Y 2012 Indication for co-evolution of *Lactobacillus johnsonii* with its hosts. BMC Microbiol 12:149. doi:10.1186/1471-2180-12-149. 22827843 PMC3503616

